# “*Every Newborn*-BIRTH” protocol: observational study validating indicators for coverage and quality of maternal and newborn health care in Bangladesh, Nepal and Tanzania

**DOI:** 10.7189/jogh.09.01902

**Published:** 2019-06

**Authors:** Louise T Day, Harriet Ruysen, Vladimir S Gordeev, Georgia R Gore-Langton, Dorothy Boggs, Simon Cousens, Sarah G Moxon, Hannah Blencowe, Angela Baschieri, Ahmed Ehsanur Rahman, Tazeen Tahsina, Sojib Bin Zaman, Tanvir Hossain, Qazi Sadeq-ur Rahman, Shafiqul Ameen, Shams El Arifeen, Ashish KC, Shree Krishna Shrestha, Naresh P KC, Dela Singh, Anjani Kumar Jha, Bijay Jha, Nisha Rana, Omkar Basnet, Elisha Joshi, Asmita Paudel, Parashu Ram Shrestha, Deepak Jha, Ram Chandra Bastola, Jagat Jeevan Ghimire, Rajendra Paudel, Nahya Salim, Donat Shamb, Karim Manji, Josephine Shabani, Kizito Shirima, Namala Mkopi, Mwifadhi Mrisho, Fatuma Manzi, Jennie Jaribu, Edward Kija, Evelyne Assenga, Rodrick Kisenge, Andrea Pembe, Claudia Hanson, Godfrey Mbaruku, Honorati Masanja, Agbessi Amouzou, Tariq Azim, Debra Jackson, Theopista John Kabuteni, Matthews Mathai, Jean-Pierre Monet, Allisyn Moran, Pavani Ram, Barbara Rawlins, Johan Ivar Sæbø, Florina Serbanescu, Lara Vaz, Nabila Zaka, Joy E Lawn

**Affiliations:** 1 Maternal, Adolescent, Reproductive & Child Health (MARCH) Centre, London School of Hygiene &Tropical Medicine (LSHTM), London, UK; 2 Maternal and Child Health Division, International Centre for Diarrhoeal Disease Research, Bangladesh (iccdr,b), Dhaka, Bangladesh; 3 Department of Women’s and Children’s Health, Uppsala University, Uppsala, Sweden; 4 Pokhara Academy of Health Science, Pokhara Ranipauwa, Nepal; 5 Department of Health Services, Ministry of Health, Kathmandu, Nepal; 6 Nepal Health Research Council, Kathmandu, Nepal; 7 Golden Community, Kathmandu, Nepal; 8 LifeLine Nepal, Kathmandu, Nepal; 9 Matri Shishu Miteri Hospital, Pokhara, Nepal; 10 Kanti Children's Hospital, Kathmandu, Nepal; 11 Department of Paediatrics and Child Health, Muhimbili University of Health and Allied Sciences, Dar Es Salaam, Tanzania; 12 Department of Health Systems, Impact Evaluation and Policy, Ifakara Health Institute, Dar es Salaam, Tanzania; 13 Public Health Sciences – Global Health – Health Systems and Policy, Karolinska Institutet, Stockholm, Sweden; 14 Institute for International Programs, Department of International Health, Johns Hopkins University, Baltimore, Maryland, USA; 15 MEAUSRE Evaluation, University of North Carolina, North Carolina, USA; 16 Knowledge Management & Implementation Research Unit, Health Section, UNICEF, New York, USA; 17 Family and Reproductive Health WHO Tanzania; 18 Centre for Maternal and Newborn Health, Liverpool School of Tropical Medicine, Liverpool, UK; 19 Department for Sexual and Reproductive Health, UNFPA, New York, USA; 20 Department of Maternal, Newborn, Child and Adolescent Health, World Health Organization, Geneva, Switzerland; 21 Office of Health, Infectious Disease and Nutrition, Bureau for Global Health, United States Agency for International Development, Washington, DC, USA; 22 Jhpiego Baltimore, Baltimore, MD, USA; 23 Department for Informatics, University of Oslo, Oslo, Norway; 24 Division of Reproductive Health, Centres for Disease Control and Prevention (CDC), Atlanta, Georgia, USA; 25 Save the Children, Washington, DC, USA; * Joint first authors; † Deceased 2 September 2018

## Abstract

**Background:**

To achieve Sustainable Development Goals and Universal Health Coverage, programmatic data are essential. The Every Newborn Action Plan, agreed by all United Nations member states and >80 development partners, includes an ambitious Measurement Improvement Roadmap. Quality of care at birth is prioritised by both Every Newborn and Ending Preventable Maternal Mortality strategies, hence metrics need to advance from health service contact alone, to content of care. As facility births increase, monitoring using routine facility data in DHIS2 has potential, yet validation research has mainly focussed on maternal recall surveys. The *Every Newborn* – Birth Indicators Research Tracking in Hospitals (EN-BIRTH) study aims to validate selected newborn and maternal indicators for routine tracking of coverage and quality of facility-based care for use at district, national and global levels.

**Methods:**

EN-BIRTH is an observational study including >20 000 facility births in three countries (Tanzania, Bangladesh and Nepal) to validate selected indicators. Direct clinical observation will be compared with facility register data and a pre-discharge maternal recall survey for indicators including: uterotonic administration, immediate newborn care, neonatal resuscitation and Kangaroo mother care. Indicators including neonatal infection management and antenatal corticosteroid administration, which cannot be easily observed, will be validated using inpatient records. Trained clinical observers in Labour/Delivery ward, Operation theatre, and Kangaroo mother care ward/areas will collect data using a tablet-based customised data capturing application. Sensitivity will be calculated for numerators of all indicators and specificity for those numerators with adequate information. Other objectives include comparison of denominator options (ie, true target population or surrogates) and quality of care analyses, especially regarding intervention timing. Barriers and enablers to routine recording and data usage will be assessed by data flow assessments, quantitative and qualitative analyses.

**Conclusions:**

To our knowledge, this is the first large, multi-country study validating facility-based routine data compared to direct observation for maternal and newborn care, designed to provide evidence to inform selection of a core list of indicators recommended for inclusion in national DHIS2. Availability and use of such data are fundamental to drive progress towards ending the annual 5.5 million preventable stillbirths, maternal and newborn deaths.

Valid data and measurement are central to achieving the Sustainable Development Goal (SDG) aspiration of “no-one left behind” [1]. In the United Nation’s Global Strategy for Women’s Children’s and Adolescent’s Health the ongoing imperative for the right to survive, is joined by a new focus on thriving, with wider transformation [2]. Progress for survival has been slowest for the 5.5 million deaths of women and babies around the time of birth each year, including an estimated 2.5 million newborns dying in the first 28 days of life, 2.6 million babies stillborn and 303 000 maternal deaths [3-5]. Most of these deaths happen to the poorest families in the poorest countries, and most are preventable [6]. Opportunity exists to save an estimated 3 million lives per year by improving quality of care at birth and care of small and sick newborns [7,8]. Based on this evidence, the *Every Newborn* Action Plan (ENAP) was launched in 2014 and endorsed by all member states in a World Health Assembly resolution [9]. The plan outlines 2030 country targets of 12 or fewer newborn deaths per 1000 live births and 12 or fewer stillbirths per 1000 total births. *Every Newborn* is closely aligned with the World Health Organization (WHO) Strategy for Ending Preventable Maternal Mortality (EPMM) [10] since both include a priority for quality of care at birth alongside the Quality, Equity, Dignity movement led by WHO, UNICEF and UNFPA in 11 countries, aiming to halve facility deaths by 2020 [11].

Accurate data are essential to drive progress towards these targets. However, at the dawn of the SDG era, most deaths around the time of birth still occur in settings with the least data on coverage and quality of care – the “inverse data law” [12]. One of five strategic objectives of *Every Newborn* is to transform measurement and use of data to track coverage and quality of care [8,9,13]. A top priority has been to develop and implement a time-limited plan to ensure required core indicators are validated and feasible to measure at scale. In support, WHO and the London School of Hygiene & Tropical Medicine (LSHTM) have coordinated an ambitious Measurement Improvement Roadmap which reviews specific measurement gaps and provides a multi-year, multi-partner pathway to define specific indicators, test validity if needed, develop tools, and promote use of data by 2020 [14-16].

Ten core indicators were prioritised as part of the *Every Newborn* multi-country consultation process including those for impact, coverage and input (**Figure 1**) [9,16,17]. This protocol relates to the coverage indicators shown in the middle of **Figure 1**. Indicators of coverage of care for all women and newborns are shaded amber, because whilst definitions are clear, content and quality of care data requires improvement. The greatest metrics gap is core coverage indicators for specific, high impact interventions, shown in red in Figure 1. The combination of core indicators for *Every Newborn* and EPMM is illustrated in Figure 2 and approximately half of these indicators are the same [10]. Validating the highest priority indicators, highlighted in red in Figure 2, is the topic of this research: all women to receive uterotonics and newborns with complications to receive neonatal resuscitation, Kangaroo mother care (KMC), treatment for possible serious infections and maternal antenatal corticosteroids (ACS)[16]. The assumed need for these interventions, likely coverage and expected prevalence is shown in the Appendix S2, Table S1 in **Online Supplementary Document**.

**Figure 1:**
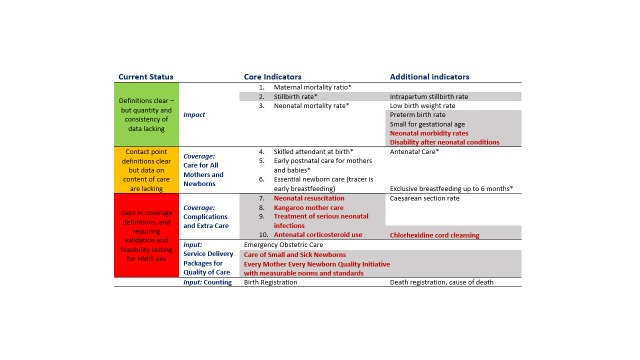
*Every Newborn* Action Plan core and additional indicators. Shaded – not currently routinely tracked at global level. Bold red – indicator requiring additional testing to inform consistent measurement. Asterisk – also SDG core or complementary indicator. Indicators disaggregated by equity such as urban/rural, income, and education. Adapted from references [9,16,17].

**Figure 2:**
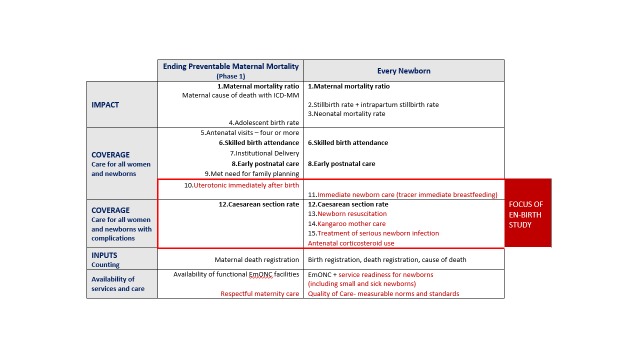
Combined priority indicator table for relevant plans: Ending Preventable Maternal Mortality and *Every Newborn* [10]. Highlighted in red with box is the priority for measurement improvement and the focus of this research.

Coverage is defined as the number of individuals receiving an intervention or service (numerator), from among the population in need of the intervention or service (denominator). To date the main source of coverage and impact data in high-burden countries has been intermittent household surveys, including: Demographic Health Survey (DHS) and Multiple Indicator Cluster Survey (MICS) [18,19]. Currently monitored coverage indicators, including antenatal care, skilled birth attendance and postnatal care, mainly measure contact points with health care services but additional indicators are required to capture effective content of care [16,20,21]. Quality of care measurement requires definitions of characteristics for both provision (eg, safety, effectiveness, timeliness, equity, completeness) and experience of care (eg, client satisfaction) [22,23]. Household survey data accuracy depends first on the woman’s interpretation of what took place at the time and second on recalling and reporting this understanding up to five years after the event. Evidence suggests that household surveys do not always accurately capture either numerator or denominator for some treatment interventions, such as pneumonia in young children [24] and events during labour [25]. In addition, since measurement of newborns with complications occur only for a subset of births (3%-15%, see Appendix S2, Table S1 in the **Online Supplementary Document**), the sample size required is higher than possible in most national DHS. Consequently, not all desired maternal and newborn intervention coverage indicators specifically relating to content and quality of care, can be captured through household surveys [16,26].

Globally more than 75% of babies are now born in facilities, and local count data from routine registers is increasingly available [27]. Whilst health-facility data can be used to track coverage more frequently than surveys, previous studies have demonstrated mixed data quality [28-30]. Health workers recording the care they deliver face many barriers in documentation [31,32]. Capturing denominators through routine data are also a major challenge. Firstly, for indicators regarding interventions for the whole population, disaggregated by equity criteria, facility births are not the “true” population denominators. Given the lack of specific and appropriate denominator data, a national health management information system (HMIS) typically use census-based data for deriving forecasts and key population calculations [28]. Secondly, the challenge is magnified if the “true” denominator for the intervention is based on clinical need, so targeted at a proportion of the total population eg, requiring treatment for possible serious bacterial infection. Measurement of the “true” denominator requires consistent and objective measure of clinical need. Yet clinical judgement and decision making, even using evidence based algorithms, is often still subjective [33,34]. Live births are often used as a proxy denominator when it is challenging to define and measure the “true” denominator. A benchmark “target coverage level” is required when proxy denominators are used, because 100% coverage is only a target for a “true” denominator. For example, the “true” denominator for Caesarean Section rate is “women in need for Caesarean section”. Because this is challenging to define and measure, the proxy denominator per 100 live births is used, but benchmarking a “target Caesarean Section rate” has proved complex [35-39]. Large inequity within countries and over- and under-provision occurring in parallel [40] highlight the problem of constructing useful indicators to measure and compare met need for complications. Therefore, an important focus of this study will be to compare various denominator options and, if using a proxy denominator to consider benchmarking.

The hierarchy of data needs (**Figure 3**) illustrates scope and granularity of data use decreases at higher levels of the health system [41]. At the point of service delivery, data are needed for individual clinical decisions and to measure the client’s perspective of care received. At facility level, aggregate data are collated to inform administrative and managerial decisions for planning and local quality improvement, mortality audit etc. At district level, data are required for planning (eg, human resources, equipment and drug availability). At national and global level, it is not possible or useful to collate all these data used at lower levels of the system. But it is crucial for accountability purposes to track a few core, standardised indicators to monitor SDGs and Universal Health Coverage at all levels – these “core indicators” are shown in the centre of the pyramid (**Figure 3**). WHO maintains a core list of 100 health indicators [42] and ENAP has prioritised 10 core indicators [9,16,17].

**Figure 3:**
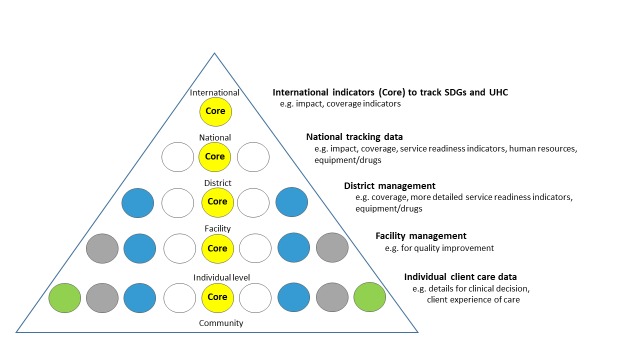
Data collection and use by level of health system. Adapted from [41].

Improvements in civil and vital registration systems are enabling a more rapid transition to more timely denominator data on births and deaths. Data systems are transitioning to increasing use of HMIS to collect, collate, analyse and report routine data from health facilities up to district and national level. This has potential to be cost-efficient and generate more frequent coverage measurements [16,27]. Electronic HMIS platforms are increasingly being applied, offering great potential to harmonize traditionally fragmented information streams [43]. One such platform, the District Health Information System, version 2 (DHIS2) [44] is now being successfully implemented in >50 countries with high mortality burdens. Infrastructure and software development advances are currently driving a transition from predominately paper-based to mixed recording systems, even at clinical data level, ie, electronic patient records will increasingly be the basis of HMIS data in low-middle income country (LMIC) contexts.

Testing indicator validity is critical to improve measurement and inform decision makers of the likely accuracy of coverage collected by household survey and/or routine facility data [20]. Comparison of the reported indicator to an external data source “gold standard” is recommended [45]. Previous validation studies have mainly focused on population-based intervention coverage indicators for use in household surveys [25,46-48]. Observational studies to determine accuracy of facility registers in high burden settings have typically focused on outcome indicators [29]. The EN-BIRTH study seeks to address current evidence gaps by testing validity of priority coverage indicators for newborn and maternal health, in facilities in three high burden country settings.

## Aim

This paper is the protocol paper for the ***E****very* ***N****ewborn*-**B**irth **I**ndicators **R**esearch **T**racking in **H**ospitals (EN-BIRTH) Study, which aims to test validity of selected newborn and maternal care health intervention indicators (coverage/ quality aspects and/or safety) in facilities (**Table 1**). This study, as part of the *Every Newborn* Measurement Improvement Roadmap, and working closely with EPMM, aims to increase the evidence base to inform selection and use of maternal and newborn indicators in national HMIS (particularly DHIS2), and global tracking.

**Table 1:** EN-BIRTH study selected indicators to be assessed for validity

Indicator	Place of care	Numerator	Denominator options
Uterotonic use for 3^rd^ stage of labour	Labour/Delivery ward, or operating Theatre	Number of women who received a uterotonic immediately after birth	- Per 100 live births (currently used denominator) - Per 100 total births
Immediate newborn care		Number of babies who breastfed immediately after birth as possible surrogate for immediate newborn care	Per 100 live births (currently used denominator)
		Number of newborns who had chlorhexidine applied to the cord stump after birth (*Bangladesh and Nepal only*)	
Newborn resuscitation		Number of newborns for whom resuscitation actions (Bag and Mask Ventilation) were initiated	**To be compared for all 4 denominators options:** - Target population requiring the specific intervention (eg, admitted to the facility with presumed infection or at risk of preterm birth as per WHO guideline) - Live births in the facility - Total births in the facility (including stillbirths) - Estimated births in the population (live or total)
Kangaroo mother care (KMC)	KMC ward/ area	Number of eligible (<2000g) newborns initiated on facility-based KMC	
Treatment of neonatal infection	Newborn or postnatal wards	Number of neonates (<28 days old) who received at least one dose of antibiotic injection*	
Antenatal corticosteroid (ACS) use	Labour/delivery ward or antenatal ward	All women giving birth in a facility who are 24-34 weeks and received at least one dose of ACS**†**	

*Specific exclusions apply to exclude other primary diagnoses eg, congenital abnormalities, preterm births <32 weeks or <1500g and neonatal encephalopathy.

**†**ACS focus is to track safety, test methods to include gestational age and relevant safety outcomes.

## Research objectives

The research questions per objective, methods and analysis are detailed in **Table 2**.

**Table 2:** EN-BIRTH study summary of research questions, data collection and analysis by objective

Research questions	Data collection method	Data analysis approach
**Objective 1 – Numerators**		
- Do registers give a valid representation of observed maternal and newborn interventions? - Do maternal recall survey questions used in household surveys capture a valid representation of the observed maternal and newborn interventions? - What is the consistency between observers?	- Observation of clinical practice (or verification from inpatient records for neonatal infections and ACS) plus video film for neonatal resuscitation (Nepal only) - Maternal recall survey (all six indicators) - Extraction from routine data sources	- Sensitivity, positive predictive value - Specificity of numerator for those with all birth denominator or clearly measurable denominator - Inter-rater reliability (Cohen’s Kappa)
**Objective 2 – Denominators**		
- How different are the coverage estimates when using alternative denominator options? - Which denominator options are feasible for use in each country HMIS?	Observation of clinical practice for measurement of “true” denominator Collection of hospital documentation for the denominator or alternative denominator options	- Descriptive statistics - Quantitative analysis with inflation factor for indicators with all-birth denominator
**Objective 3 – Content and quality of care**		
- What content of care are women and newborns observed to receive for each intervention, with focus on timing? - Which aspects of the content of care are already accurately recorded in registers? - Which aspects of the content of care are accurately recalled by women?	Observation of clinical practice (or verification from inpatient records for neonatal infections and ACS) plus video film for neonatal resuscitation (Nepal only) Maternal recall survey (all six indicators) Extraction from routine data sources	- Assessment of content/quality of care for specific aspects related to each intervention with emphasis on timing
**Objective 4 – Barriers and enablers**		
- Are some indicators recorded more completely than others? - Has routine recording changed during the time of the study? - What are the barriers and enablers to measurement of these indicators? - What are the barriers and enablers to perceived use of data regarding these indicators? - How can facility recording and flow of information into DHIS2 for these indicators be improved?	Quantitative – Register review for 12 months before and during study Qualitative FGD/IDI of study data collectors Qualitative FGD/IDI of health workers Qualitative FGD/IDI of other data users (policymakers etc) regarding data utility Process evaluation of data flow from patient level to DHIS2	- Quantitative comparison of registers applying data quality scores comparing before and after - Qualitative data for data collectors, health workers and data users - Process evaluation of data flow to DHIS2

FGD – focus group discussion, IDI – in-depth interview, DHIS2 – District Health Information System 2

**Objective 1 – Numerators:** To determine validity (accuracy) of both routine facility register and maternal recall surveys, compared to direct observation for selected maternal and newborn care interventions: uterotonics for 3rd stage labour, immediate breastfeeding, neonatal resuscitation, KMC; and, verification with patient case notes: neonatal infection management, and ACS administration (**Table 1**).

**Objective 2 – Denominators:** To compare different denominator options including proxies, and assess feasibility of their use in routine data platforms (**Table 1**), including:

- Target population requiring intervention (clinical need) in the facility (“true” denominator)
- Live births in the facility
- Total births (live births and stillbirths) in the facility
- Estimated population births (live or total): facility births *and* home births

**Objective 3 – Content /quality of care:** To evaluate different domains of coverage (eg, timing, completion rates, safety) for selected interventions (**Table 3**).

**Table 3:** EN-BIRTH study – Examples of indicator quality of care research questions, particularly regarding timing

Intervention	Research question to answer using observation data	
Uterotonic	Proportion of mothers who received oxytocin within recommended one minute after birth
Immediate breastfeeding	Proportion of babies whose breastfeeding was initiated within one hour of birth
Resuscitation	Proportion of non-breathing babies who had bag-and-mask initiated within one minute of birth
Kangaroo mother care	Proportion of babies receiving KMC, held in skin-to-skin position for 18 h or more, during the last 24 h
Neonatal infection	Proportion of cases with presumed sepsis, treated with antibiotics and for whom a blood culture result was available
Antenatal corticosteroids	Proportion of preterm labour cases who received antenatal corticosteroids according to WHO criteria for safety

**Objective 4 – Barriers and enablers:** To evaluate barriers and enablers to routine recording of selected indicators, and to explore perceived utility of these data to improve decision-making, coverage and quality of care at all levels.

## METHODS

### Study design

**Figure 4:**
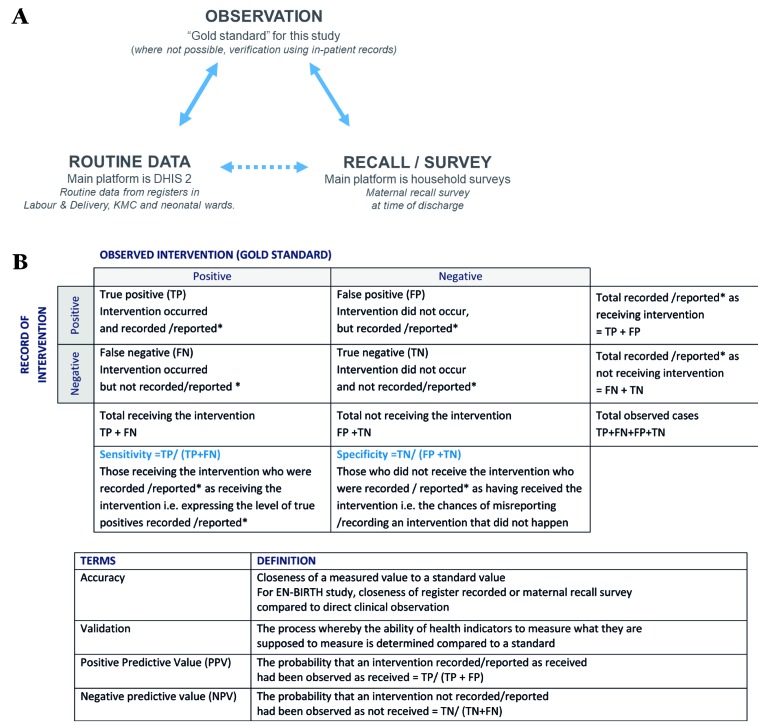
EN-BIRTH study validation and analysis approach. **Panel A.** Validation "gold standard" comparison to routine data (eg, HMIS/DHIS2) and to maternal recall survey data (eg, for household surveys). **Panel B.** Analysis for validation of sensitivity and specificity. Asterisk – recorded in facility L&D or KMC register / reported in maternal recall survey.

The EN-BIRTH study uses quantitative and qualitative methods across four objectives (**Table 2**). The validity of coverage indicators of selected maternal and newborn interventions as measured by routine facility registers and maternal recall surveys will be assessed by comparison with the “gold standard” of direct observation (**Figure 4**, panel A). Observation will be undertaken in three clinical settings (Labour/Delivery ward, Operation theatre, and KMC ward/area) by trained clinical observers. Data will be extracted from facility registers and verification of inpatient records carried out for newborns who received antibiotics for presumed infection, and for women who received ACS. Interviews to capture maternal recall will be conducted prior to discharge with all women whose births and/or their newborn’s care were observed or case notes were verified. In addition, barriers and enablers to recording of selected indicators in routine facility registers will be evaluated. Data flow into national HMIS platforms and perceived utility of data will be documented.

Research questions were informed by consultation with many *Every Newborn* stakeholders [9,17] including WHO-led Measurement Improvement Roadmap meeting [15] and EN-BIRTH Expert Advisory Group (listed as author group). More than 60 participants in an EN-BIRTH study design workshop [49] provided representation from country partners, national stakeholders, UN agencies, leading academic and professional experts in the field, governmental and non-governmental organisations, clinicians, program managers, other key experts and donors (see Appendix S1 in **Online Supplementary Document**) and contributed to development of the research protocol (**Box 1**).

Box 1 **Authorship teams for EN-BIRTH study**

**EN-BIRTH LSHTM Team:** Louise T Day, Harriet Ruysen, Vladimir S Gordeev, Georgia R Gore-Langton, Dorothy Boggs, Simon Cousens, Sarah G Moxon, Hannah Blencowe, Angela Baschieri.

**EN-BIRTH Co-PI and country teams**

**Bangladesh:** Ahmed Ehsanur Rahman, Tazeen Tahsina, Sojib Bin Zaman, Tanvir Hossain, Qazi Sadeq-ur Rahman, Shafiqul Ameen, Shams El Arifeen.

**Nepal:** Ashish KC, Shree Krishna Shrestha, Naresh P KC, Dela Singh, Anjani Kumar Jha,

Bijay Jha, Nisha Rana, Omkar Basnet, Elisha Joshi, Asmita Paudel, Parashu Ram Shrestha, Deepak Jha, Ram Chandra Bastola, Jagat Jeevan Ghimire, Rajendra Paudel.

**Tanzania:** Nahya Salim, Donat Shamba, Karim Manji, Josephine Shabani, Kizito Shirima, Namala Mkopi, Mwifadhi Mrisho, Fatuma Manzi, Jennie Jaribu, Edward Kija, Evelyne Assenga, Rodrick Kisenge, Andrea Pembe, Claudia Hanson, Godfrey Mbaruku, Honorati Masanja.

**Senior author/corresponding:** Joy E Lawn

**With the EN-BIRTH Expert Advisory group**

Agbessi Amouzou, Tariq Azim, Debra Jackson, Theopista John Kabuteni, Matthews Mathai, Jean-Pierre Monet, Allisyn Moran, Pavani Ram, Barbara Rawlins, Johan Ivar Sæbø, Florina Serbanescu, Lara Vaz, Nabila Zaka.

**On behalf of the EN-BIRTH study research design Windsor Workshop Invitees (not already names in above author groups**

AI Ayede, Simon Azariah, Anne-Marie Bergh, Elahi Chowdhury, Olive Cocoman, Patricia Coffey, Jai Das, Ashok Deorari, Mary Drake, Queen Dube, Suzanne Fournier, John Grove, Rima Jolivet, Amira Khan, Dyson Likomwa, James Litch, Goldy Mazia, Kate Milner, Indira Narayanan, Susan Niermeyer, Alfred Osoti, Sayed Rubayet, Joanna Schellenberg, Wilfred Senyoni, Gaurav Sharma, Kavita Singh, Nalini Singhal, Cally Tann, Steve Wall.

### Study settings

Tanzania, Bangladesh and Nepal were chosen as LMIC’s currently implementing the selected maternal and newborn interventions within Sub-Saharan Africa and Asia [50]. Within these countries, research centres of excellence with a strong track record in maternal and newborn health were selected: Ifakara Health Institute (IHI) and Muhimbili University of Health and Allied Sciences (MUHAS) in Tanzania, International Centre for Diarrhoeal Disease Research, Bangladesh (icddr,b); UNICEF-Nepal with Lifeline in Nepal. Criteria for selection of facilities were: providing the selected interventions in line with current WHO recommendations for improving quality of care; existing registers recording most interventions; and sufficient number of births to ensure sample size (except for ACS discussed under sample size section below).

### Study populations

**Figure 5:**
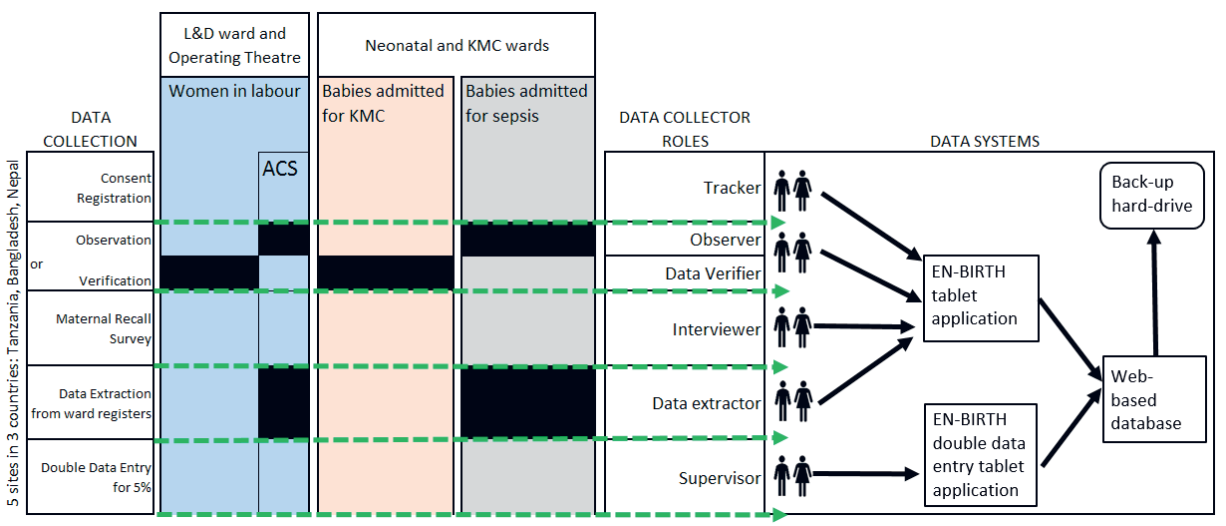
EN-BIRTH study – overview of data flow in study sites. Data Collection – "ward registers" on one line. Data collector roles revised with "Data Verifier" added. Data Systems needed "web based database" (word database was missing). ACS – antenatal corticosteroids.

Inclusion / exclusion criteria for consenting women according to data collection methods (**Figure 5**) are:

- **Observation on labour and delivery, operating theatre:** All admitted women in active labour excluding those likely to deliver immediately. Women with a prior diagnosis of intrauterine death, were also excluded to avoid further maternal distress.
- **Observation KMC ward/area:** All in-born and out-born neonates admitted for KMC.
- **Verification from inpatient records for ACS administration**: All women being observed and reported to be <34 weeks’ gestation at admission from Expected Date of Delivery (EDD).
- **Verification from inpatient records for neonatal infection cases:** All babies < 28 days old with a main diagnosis of infection (sepsis/meningitis) recorded in neonatal register or admission/discharge book. Babies will be excluded for major congenital abnormality, neonatal encephalopathy/severe asphyxia, <32 weeks’ gestation and/or admission weight <1500 grammes.
- **Maternal recall survey:** All women whose birth and/or their newborn’s KMC will be observed, or case notes verified for ACS or neonatal infection.
- **Routine register extraction:** All women whose birth and/or their newborn’s KMC will be observed.

### Sample size

Sample size was based on planned analysis for validity in objective one, by assuming 50% sensitivity ±10% precision, 50% specificity ±10% precision, with α = 0.05 and then applying the lowest previously published rates for neonatal resuscitation [51] and for KMC initiation [52,53]. Since formative data suggested >80% coverage for uterotonic administration, this indicator will be well-powered (see Appendix S2, Tables S2-3 in **Online Supplementary Document**). Hence minimal sample size is 4850 observations in each country, increased to 5390 observations to allow for a non-consent rate of 10% (**Table 4**). As expected prevalence of ACS is less than 0.5%, the resulting very large sample size was not feasible for this study [54,55]. The 5390 observations will be collected from three countries. In Tanzania and Nepal, each facility will observe this number of births, and in Bangladesh observations will take place in two facilities (**Table 4**) [4,5,56]. We anticipate a total >20 000 observed births aiming to capture at least 106 observations per intervention per country, except for ACS (**Table 4** and Appendix S2, Table S3 in **Online Supplementary Document**).

**Table 4:** EN-BIRTH study – national mortality rates, facility context and expected number of births and cases per indicator

	Context	Facilities			Sample size		
**Country**	National mortality rates*	**Name**	**Hospital type**	**Annual total births**	**Expected births in study**	**Uterotonic use†**	**Each for: resuscitation, Kangaroo mother care, neonatal infection management†**
Tanzania	MMR = 398 /100 000 NMR = 22/1000 SBR = 22/1000	Muhimbili National Hospital, Dar es Salaam	National Referral & University Teaching	9773	5390	>4310	>106
		Temeke Regional Hospital, Dar es Salaam	Regional Referral	14 655	5390	>4310	>106
**Subtotal**					**10780**	**>8620**	**>212**
Bangladesh	MMR = 176/100 000 NMR = 21/1000 SBR = 25/1000	Maternal and Child Health Training Institute (MCHTI), Dhaka	Tertiary	4488	2695	>2150	>53
		Kushtia District Hospital	Secondary	2581	2695	>2,150	>53
**Subtotal**					**5390**	**>4,310**	**>106**
Nepal	MMR = 258 /100 000 NMR = 22/1000 SBR = 18/1000	Pokhara Academy of Health Sciences	Tertiary	9427	5390	>4310	>106
**TOTAL all**				**40 924**	**21 560**	**>17 240**	**>424**

*MMR – maternal mortality ratio per 100 000 live births [5]; NMR – neonatal mortality rate per 1000 live births [54]; SBR – stillbirth rate per 1000 total births [4].

†Prevalence/incidence based on references [51-53,55,56]. More details in Appendix S2 of **Online Supplementary Document****.**

### Tool development

A formative research phase was undertaken from July – December 2016 including: health facility assessments [57], register reviews, data flow assessments, and interviews/focus group discussions (FGDs) with women, caregivers, health workers and senior facility-level staff. The results helped ensure study sites could meet inclusion criteria, achieve required sample size and informed refinement of observer checklists and data collection processes. Maternal Recall survey tools were translated into local languages and back-translated.

### Data collection software application

**Figure 6:**
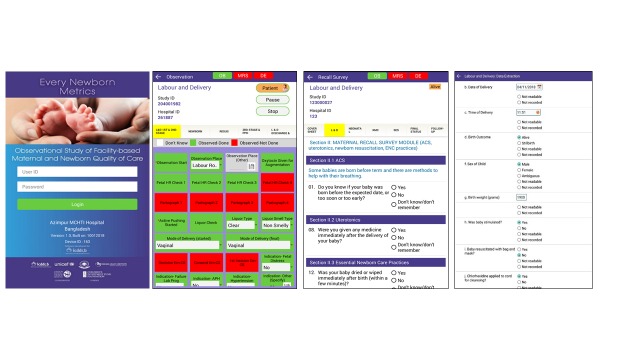
EN-BIRTH study software data collection showing examples of the tablet application screen shots.

The development of a customised tablet-based software application (Android-based) for data collection and monitoring was undertaken by the icddr,b team supported by LSHTM (**Figure 5** and **Figure 6**) [58]. The software application has different permissions for various data collector cadres (observation, verification, maternal recall survey, and data extraction) and translated into local languages where relevant. Time-stamped data will be collected using this EN-BIRTH data collection software, stored locally on the tablet, and synchronised regularly to the local central secure database server.

### Training of data collectors and supervisors

Data collector cadres include: tracker (responsible for consent, registration and assigning for observation/note verification and subsequent tracking); observer (direct observational data for assigned women and babies); interviewer (maternal recall survey interviews); data verifier/extractor (data from facility registers or case notes); and supervisor (responsible for all data collectors and quality assurance) (**Figure 5**). Observers with a clinical background (eg, nurses) will be recruited. Data collection staff will receive two weeks of training using classroom-based sessions, group activities and mock data collection within the health facility, detailed in the Data Collectors Training Handbook [58]. Observer training will include guidance on response to specific events, including managing maternal distress and when to pause data collection and assist in the care of the patient, if they perceive facility staff are responding inappropriately to a life-threatening situation. A minimum individual post-training assessment score of ≥80% is required before data collection can commence.

### Procedures according to data collection method

#### Observation (Objectives 1, 2, and 3)

Informed written consent will be obtained prior to study registration and basic demographic data collected (**Figure 5**) by the tracker. Verbal consent will be obtained from the health workers. Observers working in Labour/Delivery ward, Operating theatre and KMC ward/areas will collect direct clinical observation data. These observers will not interact with participating pregnant women, her family members or attending health workers during observation (except to respond to a life-threatening event [58]).

Observations on Labour/Delivery ward will focus on specific aspects of: 1^st^, 2^nd^ and 3^rd^ stage of labour, postpartum haemorrhage, immediate newborn care and neonatal resuscitation. Multiple parameters will be recorded to assess content/quality of care, particularly related to intervention timing. KMC observations will focus on domains of initiation, position, feeding and other treatment administered. Mother and baby outcome at discharge from hospital will be documented [58].

Additionally in Nepal for neonatal resuscitation, observation video film recording and physiological assessment will be undertaken. Information regarding these processes will be provided separately to women and informed, signed consent taken [59]. Video cameras and pulse oximeters will be placed on resuscitation tables within Labour/Delivery ward and operating theatres and research staff trained in this equipment operation and maintenance. A trained data collector will complete the observation checklist for resuscitation using the recorded video within 24 hours of birth [60,61]. If consent is subsequently withdrawn for video use, this data will be excluded, and the video deleted.

#### Verification using inpatient notes (Objectives 1, 2, and 3)

During the formative phase it was recognised that direct observation was not feasible for two of the selected interventions (neonatal infection and antenatal corticosteroids). For these interventions, data verifiers will use patient charts/case notes, drug charts, laboratory reports and other relevant routine documentation to verify intervention and quality of care measurements. Supervisors will review/search for any missing or illegible documents before confirming data not readable/ not recorded [58].

#### Maternal Recall Survey (Objectives 1, 2, and 3)

Data collectors will interview mothers whose baby’s birth or treatment is observed and/or verified prior to discharge from postnatal or KMC ward/areas The software programming of the structured questionnaires will automatically skip certain questions to minimise any risk of further emotional trauma if the mother has experienced a stillborn or neonatal death [58]. For multiple births the interview will be completed only for first-born babies. Consent will be repeated before this interview in recognition that the mother may have been in labour when she first consented to participation in this research. Consent will also be taken for repeat maternal recall surveys at different intervals after discharge, if funded for follow-up.

#### Routine register data extraction (Objectives 1, 2 and 3)

Data extractors will use routine labour/delivery registers, KMC registers and neonatal ward registers to extract participant data recorded by facility staff. If data are illegible or cannot be found, supervisors will review/search for these documents, before documenting data not readable/not recorded [58].

#### Assess barriers and enablers (Objective 4)

Mixed methods will be used to identify barriers and enablers to routine data recording and use of selected indicators (**Table 1**). Completeness and quality of existing documentation in routine registers (labour/delivery, KMC and/or neonatal) for 12 months prior to the study will be evaluated. In Bangladesh and Nepal, 100% of cases in these registers will be extracted. In the Tanzanian facilities, with a high number of births, a 20% sample randomly selected will be used for labour/delivery cases with 100% for KMC and neonatal infection cases.

Qualitative data collection tools for FGD, in-depth and key informant interviews will be informed by the MEASURE Evaluation Performance of Routine Information System Management (PRISM) conceptual framework and tools [62], including constructs for Technical, Organizational and Behavioural factors. Data will be collected from study data collectors and facility health workers. Data flow assessments will provide information on movement of data from registers, into DHIS2 and up to national level. Additionally, perceptions regarding indicators which are considered most valuable and most feasible to collect will be explored through interviews with policy makers and technical managers of DHIS2.

### Data quality monitoring

The EN-BIRTH data collection software includes skip rules, and consistency checks as well as pre-defined value ranges for some variables. Progress will be monitored by an online data dashboard, providing real-time summary tables per site, including data capture cascade for selected coverage indicators at each step; registration, consent, observation/ verification, maternal recall survey and register data extraction. A traffic light system will indicate overall progress for each indicator using pre-defined thresholds. Bi-weekly all-site calls will provide an opportunity for country teams to review and discuss progress using these data dashboards, in addition to promoting collaborative quality improvement initiatives between countries and sites.

As part of the quality assurance process, for approximately 5% of cases in each site, simultaneous supervisor observation and duplicate data verification and extraction will also be conducted using EN-BIRTH data collection software. The supervisor data will be regarded as the standard, stored in a separate database, and variability between individual data collectors estimated by calculating inter-rater reliability using Cohen’s kappa (κ) coefficient. Minimum agreement levels of ≥71% for observation and ≥91% for data extraction/case verification will be used [63].

### Data management

EN-BIRTH tablet data will be synchronised, and uploaded to an in-country central server, regularly backed-up. Raw data will be encrypted, and access restricted to country data manager who will anonymise data before data sets are pooled. Server maintenance, data management, and cleaning will be coordinated according to agreed protocols including logical and completeness checks. A unified variable code book will contain description of variable names and answer options. Qualitative data will be digitally recorded, transcribed, and translated into English. All data will be stored on password-protected computers.

### Analysis plan

Analyses will be coordinated, using a standard approach, both combining sites, and with site-specific and/or country-specific analyses. An overview of research objectives, main research questions and data analysis approach are summarised in **Table 2**. Quantitative analyses will be undertaken with Stata 15 (Stata Statistical Software: Release 1; StataCorp LLC, College Station, TX, USA).

#### Objective 1 **–** Numerator

The “gold standard” used for comparison will be direct observation of selected interventions by research observer, except for neonatal infection and ACS, where in-patient note verification will be used. Data extracted from facility routine register records and data collected during maternal recall survey will be compared with this “gold standard” separately (**Figure 4**, panel A). Accuracy of each individual coverage indicator will be assessed by constructing two-by-two tables to analyse the sensitivity and positive-predictive value of routine data (**Figure 4**, panel B). Specificity of routine data will be assessed for those indicators with true negatives and confidence intervals will be computed. “Area Under the Curve” previously used for coverage indicators validation will be used for indicators with true negatives [25,46-48,64].

#### Objective 2 **–** Denominators

Various denominator options (**Table 1**) will be compared using descriptive statistics to assess variation in estimated coverage and undertake analyses to guide benchmarking. Information on denominators will come from the EN-BIRTH data set, facility total birth data collected from facility reports, and population birth data from estimates based on census or survey and fertility rates, as used in DHIS2. For indicators with a whole population denominator (ie, uterotonics, breastfeeding) or a clearly measurable “true” denominator regarding clinical need (eg, KMC – birth weight <2000g), the inflation factor will be used. Inflation factor is the ratio of estimated routine recording-based prevalence to true (observed) population-based prevalence. It represents the magnitude of over- or under-estimation in the study setting relative to true population-based prevalence.

#### Objective 3 **–** Content/quality of care

Multiple recorded parameters will be analysed to assess measurement related to content/quality of care, particularly regarding timing of interventions and in relation to WHO Guideline recommendations (**Table 3**).

#### Objective 4 **–** Barriers and enablers

To assess barriers and enablers to indicator data recording and use, mixed methods will be used based on a framework adapted from PRISM [62] and considering other tools [65]. Quantitative analysis of routine register data collected prior to and during the study will address two research questions: (1) Are some indicators recorded more completely than others? (2) Has routine recording changed during the study time? Qualitative data from FGDs, in-depth and key informant interviews will be analysed using QSR International's NVivo 12 qualitative software (NVivo qualitative data analysis Software; QSR International Pty Ltd Version 12.1, 2018). Predetermined codes will be applied by two independent researchers, data managed into units of information covering broad categories with grouping of relevant emerging themes of importance.

## DISCUSSION

EN-BIRTH is the first large study to assess validity of newborn and maternal care indicators in routine data systems, doing so at very large scale (>20 000 observed births) across three countries with a high-burden of mortality. Previous maternal and newborn indicator validation studies have focused on testing the validity of women’s self-report method, used in population-based household surveys [25,46-48,64]. Validation of facility registers have focussed on outcome measures [29]. The EN-BIRTH study seeks to validate both routine registers and maternal recall at discharge for coverage indicators of high impact interventions. The novel software developed for this research allows detailed and precise recording of events around the time of birth, and particularly the timing of interventions. There are many studies examining quality of care at birth [66,67], and this research is not repeating that, but is focused on accuracy of routine reporting of care.

This research responds to calls from country and programme leaders for guidance on indicators for maternal and newborn services, tracking progress towards meeting national targets and Universal Health Coverage [9,17,68]. The high reporting load for many countries with multiple programmes, donors, and indicators, may result in the so-called data rich, information poor (DRIP) syndrome [69]. In addition to high reporting burden on the system, the individual midwives and doctors are responsible for recording data in multiple registers and patient records, sometimes at the expense of providing respectful quality care for women and babies. Hence a shorter list of evidence-based, indicators is required for national tracking, taking in to account validity and utility in low-resource, high-burden settings. The results of this study will inform recommendations for indicators appropriate for uptake within HMIS, and may also identify some that are not appropriate for use at higher levels of the health system (**Figure 3**). This research will also help inform improved capture and quality of data in HMIS, and especially DHIS2.

During the MDG-era, population-level surveys were the most common data source in high-burden countries, but studies consistently demonstrate challenges with maternal recall data, especially regarding details of clinical interventions [24,25,46,47,64]. For data that require medical knowledge and especially events that women may not have closely witnessed (eg, neonatal resuscitation), we expect poor maternal recall, which may reflect the lack of information given to families experiencing complications. Given continued reliance on household surveys for demographic and health data in many remote or unstable settings, we anticipate the main value of our maternal recall survey validation findings will be to contribute to the understanding of which indicators are not suitable for use in household surveys. We anticipate that if the woman does not know about the intervention at discharge from hospital, then recall later will not be useful.

A strength of this study design is the rigorous assessment of validity at scale, of facility routine data by comparison with direct observation, defined here as the “gold standard”. Another strength is a specific focus on the denominator challenge. In an era of Universal Health Coverage, with discussions surrounding scale-up of more complex care for targeted populations, the science of denominator measurement, use of proxies, and selection of benchmarks will be increasingly important. This challenge applies to denominator measurement for maternal and newborn complications (as well as other large burden conditions, notably non-communicable diseases). This study, however, is not designed to validate the denominator based on subjective assessment of clinical need (eg, requiring neonatal resuscitation). Hence, we will only be able to measure true negatives, calculate specificity, and undertake analysis of “area under the curve” for interventions with a total population or clearly defined denominator [25,46-48,64].

This research also offers a unique opportunity to examine quality of care data from >20 000 births and assess to what extent we can accurately capture specific components including content and timing of selected interventions. Although multiple specific aspects of care may be measured locally to drive quality of care improvement at facility level, here we will focus on quality of care indicators that may be useful at district or national levels of the health system. Timing of interventions is a critical marker of quality of care, since delays are a matter of life or death: a woman may die in hours, a baby in minutes. Moreover, the sequence of interventions is complex and even concurrent (eg, how often is the correct dose of uterotonic given <1 minute after birth to prevent a woman bleeding from postpartum haemorrhage; How soon is bag-and-mask ventilation initiated for a baby who is not breathing; How many hours each day is a baby kept in KMC position). The time-stamped design of EN-BIRTH data collection software will permit analysis of such sequences.

Whilst direct observation is considered the “gold standard”, data collectors might miss interventions, with concurrent actions at birth, especially in an emergency. We will limit potential recording bias by using observers with health backgrounds who are familiar with the procedures under observation [70-72]. EN-BIRTH data will also be directly on the tablet software to allow fast data capture. The study also presents several ethical challenges including the dilemma of observing a life-threatening situation without appropriate response from facility staff, and gaining informed consent during labour [58]. The clinically trained observers will have underlying familiarity of hospital environments, experience to uphold study protocols correctly [70] and experience in maintaining participant confidentiality. Training and processes will be put in place to take account of professional and legal duty of care.

The “Hawthorne effect” describes the phenomenon when a research participant’s behavior is altered as a consequence of being studied or observed, and can be a source of bias in observational research [73]. Within this study, it is possible that clinical observers’ presence will influence health workers to change their approach to care and routine register data. However, there is some evidence to suggest that sustained contact with participants (as with this study) may mitigate altered behaviors in health care settings [74]. To assess this bias, we will analyze changes in register data completeness and quality before and during the study.

Although the EN-BIRTH study is not powered to validate an ACS administration indicator, this will be included. Current WHO guidelines provide strong recommendation for the provision of a single course of ACS for any woman at risk of imminent preterm birth (24-34 weeks of gestation) provided the following criteria are met: 1) accurate assessment of gestational age; 2) no evidence of maternal infection; 3) preterm birth is considered imminent; 4) available adequate childbirth and newborn care services [75]. EN-BIRTH study sites were assessed in accordance with these WHO guidelines. The Antenatal Corticosteroid Trial (ACT) evaluated use of ACS at lower levels of the health system, with half of study births in home settings and care often provided by traditional birth attendants [76]. ACT reported an adverse outcome risk particularly in cases where ACS administration was after 34 weeks and outlines important challenges for measurement of gestational age, and assessment of maternal infection. This demonstrated need for robust data and further evidence in such settings, along with the imperative of ensuring safety and effectiveness, make measurement of ACS coverage and outcomes essential. Therefore, the EN-BIRTH study ACS analysis will focus on assessing relevant documentation to report the current ACS administration practice, compared with WHO safety criteria [75].

Given the importance of the neonatal period in terms of risk and prevention of long-term adverse child development outcomes, we plan a five-year follow-up for EN-BIRTH study recruited children who received basic neonatal interventions [77]. The ***E****very* ***N****ewborn* – **S**implified **M**easurement **I**ntegrating **L**ongitudinal **N**eurodevelopment & **G**rowth (EN-SMILING) aims to detect child development outcomes as early as possible for referral to services, and to improve routine measurement of child development outcomes in programme settings.

The EN-BIRTH study is richer through active involvement of experts and policymakers from the EN-BIRTH Expert Advisory Group, *Every Newborn* implementation community, EPMM, UN Agencies including WHO, UNICEF and UNFPA as well as many partners and donors. In further support of this goal, each of the three countries have National Advisory Committees who will actively participate in the research process and support uptake of findings. Results will also be published in peer reviewed journals and disseminated with all relevant audiences. Following EN-BIRTH study validity testing, an important next step will be to evaluate feasibility of a short-list of indicators at different levels of the health system.

Most of the 5.5 million deaths around the time of birth [3] still occur in settings with the least data. Household surveys remain a key data source in the poorest countries, and Every Newborn is also involved in a multi-site study, EN-INDEPTH, to assess and improve these data [78]. Data improvement is fundamental for monitoring more rapid progress towards meeting global and national mortality targets, and in achieving Universal Health Coverage for all women and newborns [15]. With ongoing investment in electronic data platforms (including DHIS2) and increasing country demand for evidence-based indicators, we anticipate that these results will advance availability and use of data to change coverage, quality and equity, to help end preventable maternal and newborn mortality, as well as stillbirths.

## Additional material

Online Supplementary Document
