# Supplementary material for: “Every Newborn-BIRTH” protocol: observational study validating indicators for coverage and quality of maternal and newborn health care in Bangladesh, Nepal and Tanzania
Source: J Glob Health. 2019 Mar 7;9(1):010902. doi: 10.7189/jogh.09.01902 (PMC6406050; doi:10.7189/jogh.09.01902)
Supplement: Online Supplementary Document [file jogh-09-010902-s001.pdf]

J Glob Health 2019; 9: 010902

"*Every Newborn* Birth Indicator Research Tracking in Hospitals" (EN-BIRTH) Observational Study Protocol in Tanzania, Bangladesh and Nepal: Validating indicators for coverage and quality of maternal and newborn health care

Supplementary On Line Materials

Contents

Annex 1: Authorship Group Information.......... 2

Annex 2: Data regarding Sample Size Calculations 6

Annex 3: Summary of ethics approvals 8

Annex 1: Authorship Group Information

EN-BIRTH Expert Advisory Group members

Institution	Representative
Centres for Disease Control and Prevention	Florina Serbanescu
Countdown/International Confederation of Midwives/Johns Hopkins University	Agbessi Amouzou
District Health Information System 2	Johan Ivar Sæbø
International Federation of Gynecology and Obstetrics /Royal College of Obstetricians and Gynaecologists/Liverpool School of Tropical Medicine	Matthews Mathai
Maternal Child Survival Program	Barbara Rawlins
MEASURE Evaluation	Tariq Azim
United Nations Population Fund	Jean-Pierre Monet
United Nations Children's Fund	Nabila Zaka
United Nations Children's Fund	Debra Jackson
United States Agency for International Development	Pavani Ram
World Health Organization	Allisyn Moran
Save the Children	Lara Vaz
World Health Organization, Tanzania	Dr Theopista John

EN-BIRTH study Research Design Windsor Workshop Participants (not already named in above author groups)

Institution	Representative	Named in authorship group
American Academy of Pediatrics	Susan Niermeyer	Yes
Aga Khan University	Jai Das	Yes
Applying Science to Strengthen and Improve Systems	Tamar Chitashvili	No
The Children's Investment Fund Foundation	Suzanne Fournier	Yes
The Children's Investment Fund Foundation	Ly Nguyen	No
Global Alliance to Prevent Prematurity and Stillbirth	James A. Litch	Yes
Georgetown University	Indira Narayanan	Yes
International Centre for Diarrhoeal Disease Research, Bangladesh	Elahi Chowdhury	Yes
Ipas Bangladesh	Sayed Rubayet	Yes
JHPIEGO	Mary Drake	No
Liverpool School of Tropical Medicine	Juan Dewez	No
London School of Hygiene and Tropical Medicine	Amanda Cleeve	No
London School of Hygiene and Tropical Medicine	Maya Kohli-Lynch	No
London School of Hygiene and Tropical Medicine	Kate Milner	Yes
London School of Hygiene and Tropical Medicine (IDEAS)	Joanna Schellenberg	Yes
London School of Hygiene and Tropical Medicine	Gaurav Sharma	Yes
London School of Hygiene and Tropical Medicine	Cally Tann	Yes
Maternal Child Survival Program	Goldy Mazia	Yes
Maternal Health Task Force	Rima Jolivet	Yes
MEASURE Evaluation	Kavita Singh	Yes
PATH	Patricia S. Coffey	Yes
Progressive Development Initiative	Simon Azariah	Yes
Save the Children	Mary Kinney	No
Save the Children	Dyson Likomwa	Yes
Save the Children	Steve Wall	Yes
SickKids	Amira Khan	Yes
Ministry of Health, Tanzania	Mary Azayo	No
United Nations Population Fund	Luc De Bernis	No
United Nations Population Fund	Michel Brun	No
United Nations Population Fund/International Confederation of Midwives	Petra Ten Hoope Bender	No
University of Calgary	Nalini Singhal	Yes
University of Ibadan	Adejumoke Idowu Ayede	Yes
University of Malawi	Queen Dube	Yes
University of Nairobi	Alfred Osoti	Yes
University of Oslo	Wilfred Senyoni	Yes
University of Oslo	Ola Titlestad	No
University of Pretoria	Anne-Marie Bergh	Yes
United States Agency for International Development	Troy Jacobs	No
World Health Organization/ Every Newborn Action Plan	Olive Cocoman	Yes
World Health Organization	Jon Simon	No
World Health Organization	John Grove	Yes
Collaborating Centre for Education & Research in Newborn Care, World Health Organization	Ashok Deorari	Yes

Working Group Participants EN-BIRTH study Research Design Windsor Workshop

WORKING GROUP	FACILITATORS	PARTICIPANTS
**DAY 1, SESSION A**		
1. Uterotonics	Allisyn Moran & Rima Jolivet	Georgia Gore-Langton, Gaurav Sharma, Elahi Chowdhury, Matthews Mathai, Luc De Bernis, Agbessi Amouzou, Honorati Masanja, Wilfred Senyoni
2. KMC	Goldy Mazia & Sarah Moxon	Theopista John, Simon Cousens, Suzanne Fournier, Lara Vaz, Kate Milner, Shams El Arifeen, Ashok Deorari, Queen Dube, Dyson Likomwa, Anne-Marie Bergh, Amanda Cleeve, Debra Jackson
3. Resuscitation	KC Ashish & Harriet Ruysen	Angela Baschieri, Maya Kohli Lynch, Tariq Azim, Mary Azayo, Michel Brun, Trish Coffey, Mary Drake, Vladimir Gordeev, Indira Narayanan, Barbara Rawlins, Kavita Singh, Tazeen Tahsina, Ola Titlestad, Ly Nguyen
4. Treatment of pSBI/neonatal infections	Troy Jacobs & Steve Wall	Rubayet Sayed, Nalini Singhal, Tamar Chitashvili, Juan Dewez, Ehsan Rahman, Jon Simon, Amira Khan , A I Ayede, Jai Das, Joanna Schellenberg, Hannah Blencowe, Debra Sitrin (remotely).
5.Antenatal corticosteroids	Alfred Osoti, Jim Litch, Joy Lawn	Cally Tann, Dorothy Boggs, Florina Sebabescu, Oliva Cocoman
**DAY 2, SESSION B**		
1. Denominator options for testing.	Hannah Blencowe, Simon Cousens & Angela Baschieri	Vladimir Gordeev, Joy Lawn, Michel Brun, Tamar Chitashvili, Mary Drake, Tazeen Tahsina, Ola Titlestad, Matthews Mathai, Luc De Bernis, Florina Sebanescu, Jai Das, Alfred Osoti
2. Training observers	Barbara Rawlins & Tariq Azim	Mary Azayo, I. Ayede, Amanda Cleeve, Goldie Mazia, Indira Narayanan
3. Filming, including ethics, methods, and analysis	Allisyn Moran & KC Ashish	Gaurav Sharma, Nalini Singhal, Shams El Arifeen, Joanna Schellenberg, Queen Dube.
4. Pre-discharge interviews to assess maternal recall	Agbessi Amouzou & Kavita Singh	Harriet Ruysen, Amira Khan Rubayet Sayed, Theopista Johns,
5.Qualitative health-worker interviews to assess barriers and facilitators to data recording	Lara Vaz & Anne Marie Bergh	Susan Niermeyer, Juan Dewez, Sarah Moxon
6.Knowledge management/ dissemination	Olive Cocoman & Dorothy Boggs	Georgia Gore-Langton, Troy Jacobs, Wilfred Senyoni, Ashok Deorari, Debra Jackson
**DAY 2, SESSION C**		
1.Data collection, database design and checks, plan for analysis	Joanna Schellenberg, Simon Cousens & Shams El-Arifeen	Vladimir Gordeev, Angela Baschieri, Ashish Kc, Susan Niermeyer, Tariq Azim, Tamar Chitashvili, Honorati Masanja.
2.Service readiness for small & sick newborns (linked to EmOC)	Sarah Moxon & Queen Dube	Georgia Gore-Langton, Lara Vaz, Juan Dewez, Ehsan Rahman, Queen Dube, Nalini Singhal, Anne-Marie Bergh, Michel Brun, Mary Azayo, Steve Wall, Rubayet Sayet, Indira Narayanan, Al Ayede and Goldy Mazia. In addition Neal Russell, Ashok Deorari and Hannah Blencowe (remotely)
3. Perinatal audit (linked to MDSR)	Matthews Mathai, Florina Sebanescu	Theophista Johns, Luc De Burnis, Jon Simons

4. DHIS platforms and linking to these from the start	Ola Titlestad & Wilfred Senyoni	Steve Wall, Agbessi Amouzou, Rima Jolivet, Hattie Ruysen, Olive Cocoman, Theopista Johns.
5.Birth certificate registration for all facility births	Debra Jackson & Joy Lawn	Tazeen Tahsina, Dorothy Boggs
6.Birth weight measurement for all facility births	Hannah Blencowe & Ashok Deorari	Gaurav Sharma

Annex 2: Data regarding Sample Size Calculations

Supplementary Table 1: Estimated prevalence of coverage of selected maternal/newborn interventions from global literature

INDICATOR	ASSUMED NEED	LIKELY COVERAGE OF INTERVENTION	EXPECTED PREVALENCE OF INTERVENTION PRACTICE
**Uterotonic use for 3rd stage of labour**	All women who give birth.	High for facility births	High (target all births, high coverage).
**Antenatal corticosteroid (ACS) use**	Preterm prevalence in Tanzania (11%) and Bangladesh (14%). Approximately 25% of preterm births are <34 weeks gestational age [1].	Low (e.g. IQR 30-68%) [2]	Approximately 0.4-0.5% of all births.
**Newborn resuscitation**	Approximately 3% of births require resuscitation with bag and mask [3].	HBB wide scale up in both Tanzania and Bangladesh. Moderate - approximately 50% of facility born neonates receive resuscitation when needed.	Approximately 1-2% of all births.
**Kangaroo mother care (KMC)**	Approximately 20% of live births in Bangladesh and 10% in Tanzania are <2,000g.	Tanzania low to moderate - approximately 20%. Bangladesh - very low approximately <10% [4].	Tanzania - approximately 2% of live births Bangladesh - approximately 2% of live births.
**Treatment of neonatal possible serious bacterial infection (PSBI)**	Approximately 7% of live births during neonatal period [5]. >70% of deaths attributed to neonatal infections are in first week [6].	Tanzania - moderate as wide scale up of IMNCI - approximately 40%. Bangladesh - low/moderate - approximately 40% of those in facilities.	Tanzania - approximately 3% of live births. Bangladesh- approximately 3% of live births.

References

1 Blencowe H, Cousens S, Oestergaard MZ, Chou D, Moller A-B, Narwal R, et al. National, regional, and worldwide estimates of preterm birth rates in the year 2010 with time trends since 1990 for selected countries: a systematic analysis and implications. The Lancet. 2012;379:2162-72.

2 Vogel JP, Souza JP, Gülmezoglu AM, Mori R, Lumbiganon P, Qureshi Z, et al. Use of antenatal corticosteroids and tocolytic drugs in preterm births in 29 countries: an analysis of the WHO Multicountry Survey on Maternal and Newborn Health. The Lancet. 2014;384:1869-77.

3 Lee AC, Cousens S, Wall SN, Niermeyer S, Darmstadt GL, Carlo WA, et al. Neonatal resuscitation and immediate newborn assessment and stimulation for the prevention of neonatal deaths: a systematic review, meta-analysis and Delphi estimation of mortality effect. BMC public health. 2011;11:S12.

4 Vesel L, Bergh A-M, Kerber KJ, Valsangkar B, Mazia G, Moxon SG, et al. Kangaroo mother care: a multi-country analysis of health system bottlenecks and potential solutions. BMC pregnancy and childbirth. 2015;15:S5.

5 Seale AC, Blencowe H, Manu AA, Nair H, Bahl R, Qazi SA, et al. Estimates of possible severe bacterial infection in neonates in sub-Saharan Africa, south Asia, and Latin America for 2012: a systematic review and meta-analysis. The Lancet infectious diseases. 2014;14:731-41.

6 Oza S, Lawn JE, Hogan DR, Mathers C, Cousens SN. Neonatal cause-of-death estimates for the early and late neonatal periods for 194 countries: 2000–2013. Bulletin of the World Health Organization. 2015;93:19-28.

Supplementary Table 2: Formative Phase Data by country and facility site used to inform EN-BIRTH study sample size

Country	Facility	Average annual births	uterotonic	Resus	KMC	Neonatal infection treatment
Tanzania	Muhimbili National Hospital, Dar es Salaam	9,773	n=9,773 (100%)	n=560 (5.7%)	432 (4.4%)	>80% of neonates admitted to neonatal ward treated with antibiotics
	Temeke Regional Hospital Dar es Salaam	14,655	n=14,655 (100%)	n=488 (3.3%)	564 (3.8%)	651 newborns admitted to neonatal ward
Bangladesh	Maternal and Child Health Training Institute (MCHTI) Dhaka	4,488	n=3,270 (73%)	n=224 (5%)	50 (1.1%)	-
	Kushtia District Hospital (DH) Kushtia	2,581	n=2,064 (80%)	n=130 (5%)	120 (4.6%)	472
Nepal	Pokhara Academy of Health Sciences Pokhara	9,427	n=9377 (99%)	n=235 (2.5%)	250 (2.6%)	>80% of neonates admitted to neonatal ward treated with antibiotics

Note: ACS recording is more limited and data are not shown in this table

Source: Data collected in formative phase from Facility reports, 2016

Supplementary Table 3: EN-BIRTH Sample size calculation

INDICATOR	ASSUMED PREVALENCE	EXPECTED SENSITIVITY	EXPECTED SPECIFICITY	DESIRED PRECISION	CONFIDENCE LEVEL	SENSITIVITY SAMPLE SIZE	+10% NON-CONSENT	SPECIFICITY SAMPLE SIZE	+10% NON-CONSENT
**Uterotonic Use**	0.80	0.50	0.50	0.10	95%	122	136	486	540
**ACS**	0.005	0.50	0.50	0.10	95%	19401	21556	98	109
**Newborn resuscitation**	0.02	0.50	0.50	0.10	95%	**4851**	**5390**	99	110
**KMC**	0.02	0.50	0.50	0.10	95%	**4851**	**5390**	99	110
**Treatment of neonatal infection**	0.03	0.50	0.50	0.10	95%	3234	3593	101	113

Annex 3: Summary of ethics approvals

Supplementary Table 3: Summary of EN-BIRTH IRB approvals with dates and references numbers

EN-BIRTH IRB	APPROVAL DATE	REFERENCE NUMBER
**LSHTM**		
LSHTM IRB	3 October 2016	11780
**TANZANIA**		
IHI IRB	20 October 2016	032-2016
MUHAS IRB	21 October 2016	2016-10-21/AEC/Vol.XI/310
National institute for medical research IRB	20 January 2017	NIMR/HQ/R.8a/Vol.IX/2394
**NEPAL**		
Nepal health research council	8 August 2016	187 /2O16
**BANGLADESH**		
ICDDR,B research review committee	11 August 2016	PR-16055
ICDDR,B ethical review committee	14 November 2016	PR-16055
